# Genetic Diversity and Population Structure of Maize Inbred Lines with Varying Levels of Resistance to *Striga hermonthica* Using Agronomic Trait-Based and SNP Markers

**DOI:** 10.3390/plants9091223

**Published:** 2020-09-17

**Authors:** Adekemi Stanley, Abebe Menkir, Agre Paterne, Beatrice Ifie, Pangirayi Tongoona, Nnanna Unachukwu, Silvestro Meseka, Wende Mengesha, Melaku Gedil

**Affiliations:** 1 West Africa Centre for Crop Improvement University of Ghana, Legon PMB 30, Ghana; estanley@wacci.ug.edu.gh (A.S.); bifie@wacci.ug.edu.gh (B.I.); ptongoona@wacci.ug.edu.gh (P.T.); 2 International Institute of Tropical Agriculture (IITA), Ibadan 200001, Nigeria; P.Agre@cgiar.org (A.P.); N.Unachukwu@cgiar.org (N.U.); S.Meseka@cgiar.org (S.M.); w.mengesha@cgiar.org (W.M.); m.gedil@cgiar.org (M.G.)

**Keywords:** genetic diversity, genotyping-by-sequencing, maize, population structure, SNP markers, *Strigahermonthica*

## Abstract

*Striga hermonthica* is a serious biotic stress limiting maize production in sub-Saharan Africa. The limited information on the patterns of genetic diversity among maize inbred lines derived from source germplasm with mixed genetic backgrounds limits the development of inbred lines, hybrids, and synthetics with durable resistance to *S. hermonthica*. This study was conducted to assess the level of genetic diversity in a panel of 150 diverse maize inbred lines using agronomic and molecular data and also to infer the population structure among the inbred lines. Ten *Striga*-resistance-related traits were used for the phenotypic characterization, and 16,735 high-quality single-nucleotide polymorphisms (SNPs), identified by genotyping-by-sequencing (GBS), were used for molecular diversity. The phenotypic and molecular hierarchical cluster analyses grouped the inbred lines into five clusters, respectively. However, the grouping patterns between the phenotypic and molecular hierarchical cluster analyses were inconsistent due to non-overlapping information between the phenotypic and molecular data. The correlation between the phenotypic and molecular diversity matrices was very low (0.001), which is in agreement with the inconsistencies observed between the clusters formed by the phenotypic and molecular diversity analyses. The joint phenotypic and genotypic diversity matrices grouped the inbred lines into three groups based on their reaction patterns to *S. hermonthica,* and this was able to exploit a broad estimate of the actual diversity among the inbred lines. The joint analysis shows an invaluable insight for measuring genetic diversity in the evaluated materials. The result indicates that wide genetic variability exists among the inbred lines and that the joint diversity analysis can be utilized to reliably assign the inbred lines into heterotic groups and also to enhance the level of resistance to *Striga* in new maize varieties.

## 1. Introduction

Maize (*Zea mays* L.) is an important cereal crop playing a crucial role in global food security and as a source of income for smallholder farmers in sub-Saharan Africa (SSA). It is referred to as the queen of cereals because of its high yield potential, ease in processing, and low cost [1]. Maize is a major source of food and livelihood for over 900 million people in Africa [2]. Its production exceeds that of rice and wheat [3], and it holds a unique position in world agriculture. Maize has enormous genetic diversity due to its prolonged selection [4,5,6], and it has become a model crop for major cereals [7] because its genome harbors tremendous phenotypic and molecular diversity [8]. Its molecular diversity is about fivefold higher than other domesticated crops [8,9].

Maize has the potential to alleviate food insecurity in SSA, but several biotic and abiotic stresses hamper its production. These stresses account for over 50% reduction in grain yield estimated at over USD 7 billion resulting in hunger, malnutrition, and food scarcity [10]. *Striga* poses severe threat to cereal production in SSA. Among the more than 40 known *Striga* species worldwide, *Striga hermonthica* [Del.] Benth is the most destructive, harmful, and widespread in SSA, causing significant yield loss in cereals [11,12]. *S. hermonthica* is a root hemiparasite plant that parasitizes its host and extracts water and essential nutrients from it, resulting in stunting, wilting, chlorosis, reduction in yield, and death of the host. Grain yield losses due to *S. hermonthica* can reach up to 100% in susceptible maize cultivars under severe field infestation [13]. The use of resistant varieties, in combination with other control measures, including fertilizer and rotation, is considered a viable approach to combat the menace caused by this parasitic plant. For decades, maize breeders at the International Institute of Tropical Agriculture (IITA) have developed *S. hermonthica* tolerant and resistant lines. These lines have been developed from populations, composites, a backcross containing *Z. diploperennis* and synthetics that have been improved for field resistance to *S. hermonthica* using recurrent selection schemes [14,15]. These source populations were developed from mixtures of diverse maize germplasm without due regard to their heterotic patterns. Assessing the genetic diversity of these inbred lines using agronomic based-traits and molecular data will facilitate the selection of potential parental lines for use in developing desirable bi-parental source populations and also superior hybrids and synthetics with durable resistance to *S. hermonthica* [16,17].

Genetic diversity is the foundation for crop improvement, and it plays an important role in breeding programs [18]. Information on genetic diversity and relatedness among maize inbred lines have been useful in selecting parental combinations for developing superior hybrids and for assigning inbred lines into heterotic groups [19,20,21]. Population structure analysis also provides a valuable understanding of genetic diversity and serves as a guide for selection [22]. Many assessment methods, including morphological markers, heterosis, pedigree data, and molecular markers, have been used to estimate genetic diversity in plants [23,24]. Morphological markers have been widely used to assess genetic diversity because they are cheap, rapid, and easy to measure [25]. However, they are highly influenced by the environment and several other factors limit their ability to estimate genetic diversity [16]. Molecular markers are a useful tool for assessing genetic diversity because they are stable, polymorphic, readily available in the genome, and are not sensitive to environmental factors [26]. Several types of molecular markers have been used to assess genetic diversity and group maize inbred lines into heterotic groups [27,28,29]. Among the numerous types of molecular markers used are single nucleotide polymorphism (SNP) markers. SNPs are mostly used because of their stability and abundance in the genome [30]. They have been widely used in plants to evaluate genetic diversity, construct linkage maps, and association analysis [31,32]. However, studies have shown that joint analysis of molecular and morphological data provides in-depth insight into population structure and genetic diversity, and this has been used in different crops [33,34,35,36,37]. Belalia et al. [38] demonstrated the usefulness of this method in maize by assessing the genetic diversity of 56 populations using 14 agro-morphological traits and 18 simple sequence repeat (SSR) markers. Both marker systems revealed significant genetic diversity in the maize populations. The objectives of this study were, therefore, to assess the genetic structure and genetic diversity in a panel of tropical maize inbred lines using agronomic traits recorded under *Striga*-infested conditions and molecular marker (SNPs).

## 2. Materials and Methods

### 2.1. Plant Materials

A total of 150 inbred lines at S_7_ to S_9_ stages of inbreeding having diverse reactions to *S. hermonthica* were randomly selected from a large number of lines with different genetic backgrounds and used in the present study. The inbred lines were derived from diverse source populations (Table 1). Ten inbred lines with known resistance (9450), tolerance (5012, 1393, 1368, 4001, 9030, 9071, KU1414-SR, and MMB90) and susceptibility (5057) reactions to *S. hermonthica* were included as benchmarks to assess the performance of the lines.

**Phenotypic Evaluation**: The inbred lines were evaluated under *Striga*-infested conditions at Abuja (9°15′ N, 7°20′ E; 490 m asl) and Mokwa (9°21′ N, 5°10′ E; 210 m asl) in Nigeria during the main rainy seasons of 2017 and 2018. The lines were arranged in a 15 × 10 alpha lattice design with two replications and planted in strips. Within each strip, an inbred line was planted in a 4 m long row, with 0.75 m inter-row spacing and 0.25 m intra-row spacing.

The field was treated with ethylene gas two weeks before planting to remove *S. hermonthica* seeds from the soil through suicidal germination. *S. hermonthica* seeds used for artificial infestation of the infested blocks were collected from farmers’ sorghum fields in the previous planting year. Two maize seeds were planted in a 6 cm deep hole injected with 8.5 g of sand mixed with *Striga* seeds. The mixture contains approximately 3000 germinable *Striga* seeds. Two weeks after planting, all maize plants were thinned to one plant per hill to attain a population density of 53,333 plant ha^−1^. Fertilizer was applied at the rate of 30 kg/ha of Nitrogen, 60 kg/ha each of phosphorus and potassium at planting, and an additional 30 kg/ha nitrogen was applied four weeks later. Weeds other than *Striga* were removed from plots manually throughout the planting season.

### 2.2. Trait Measurements

Data recorded under *Striga*-infested conditions include: plant stand, anthesis and silking days, plant height, ear aspect, and grain yield (Supplementary Table S1). The total number of plants was counted in each plot immediately after thinning. Days to anthesis and silking were recorded as the number of days from planting to when 50% of the plants in a plot had anthers shedding pollen and showing emerged silks, respectively. Anthesis–silking interval was calculated as the interval in days between dates of silking and anthesis. Plant height was measured in centimeters as the distance from the base of the plant to the height of the first tassel branch. Ear aspect was scored on a scale of 1 to 5, where 1 = clean, uniform, large and well-filled ears, and 5 = rotten, variable, poorly filled, and small ears. All ears harvested from each plot were shelled to determine per cent moisture, which was used to determine grain yield adjusted to 15% moisture under infested conditions. *Striga* damage rating was visually rated in each infested row at 8 and 10 weeks after planting using a scale of 1 to 9, where 1 = no visible host plant damage symptom, and 9 = all leaves completely scorched, resulting in premature death [39]. Additionally, the number of emerged *S. hermonthica* plants was counted in each infested row at 8 and 10 weeks after planting.

### 2.3. Genotyping

DNA was extracted from young leaves of each inbred line using a modified cetyltrimethylammonium bromide (CTAB) protocol [40]. Purified DNA was sent to Elshire group facility in New Zealand for genotyping-by-sequencing [41]. Genomic DNA was digested with the restriction enzyme *ApeK1*, and genotyping-by-sequencing libraries were constructed in 96-plex and sequenced on Illumia HiSeq2500 [41]. Raw flow cell output was processed to genotype calls using the trait analysis by association, evolution and linkage (TASSEL)-GBS pipeline [42]. Reads and tags found in each sequencing result were aligned to the *Zea mays* L. genome reference, version *AGPV4* (B73 RefGen v4 assembly). 

## 3. Statistical Analysis

### 3.1. Phenotypic Analysis

Analyses of variance combined across the four year–location combinations were computed for all traits measured under *Striga* infested conditions based on mixed-model analysis with the restricted maximum likelihood procedure in SAS version 9.4 [43]. In this analysis, environments, replication (environment), and block (replication × environment) were considered as random effects. Heritability estimates and Least-squares means (Lsmeans) were generated from the combined analysis. The Lsmeans generated from the combined analysis was used for principal component analysis in FactorMiner and missMDA R packages [44]. The optimal number of factors to be retained was determined using the principle of Peres–Nero [45] while the dimdesc function implemented in missMDA was used to assess the contribution of each trait. Using the most discriminant variable, Gower’s dissimilarity matrix was generated using cluster R package, and the count variables were scaled. The final Hierarchical cluster analysis was performed using ward.D2 method in cluster R package [46]. Correlation among the different phenotypic variables was performed using the R software, and results were displayed as a heatmap.

### 3.2. Quality Control and Genotypic Analysis

Over 560,000 SNPs were received from the Elshire group in New Zealand. To determine the quality of the data, quality control (Q.C.) was performed to retain only bi-allelic sites, SNPs with minor allele frequency (MAF) less than 5% and missing data more than 10% were removed from the data set. In addition, SNPs with high linkage disequilibrium (L.D) were pruned using the indep-pairwise function implemented in PLINK (SNP window size: 50, SNPs shifted per step: 5, r2 thresholds: 0.5) [47]. Based on the above parameter, 16,735 SNP markers were retained for further analysis. Summary statistics, including minor allele frequency (MAF), polymorphic information content (PIC), heterozygosity, and gene diversity, were estimated using”--freq” and “--hardy” functions implemented in plink [47]. Population structure analysis was performed through ADMIXTURE [46] using a cross-validation error (*k*) ranging from *k* = 2 to *k* = 10 [48,49]. The optimal number of clusters was inferred using k-means analysis after varying the possible number of clusters from 2 to 50 using the Bayesian information criterion (BIC). A discriminant analysis of principal components (DAPC) was carried out using the adegenet R package [50]. Membership probabilities of the individuals for the different groups were estimated using a cut-off value of 80% suggested through the DAPC. A pairwise genetic distance matrix was calculated using identity-by-state (IBS). A Ward’s minimum variance hierarchical cluster dendrogram was then generated from the genetic distance matrix using the analyses of phylogenetics and evolution (ape) R package [51,52].

### 3.3. Joint Analysis of Agronomic-Based and Molecular Data

Genetic groups were defined using a combination of the agronomic trait-based and genotypic dissimilarity matrices. The joint matrix was generated by the summing the genotypic and agronomic trait-based dissimilarity matrices. Similarities between the hierarchical clusters generated from the agronomic trait-based and genotypic data were accessed using the tanglegram function implemented in the dendextend R package [53]. The relationship between the dissimilarity matrices of the agronomic trait-based matrix under *Striga* infestation, genotypic and joint distance matrices were assessed using a Mantel test based on the Monte-Carlo method with 9999 permutations. 

## 4. Results

### 4.1. Diversity Based on Agronomic Traits

In the combined analyses of variance, differences among environments and inbred lines were significant for all traits measured under *Striga* infestation except for ear aspect (Supplementary Table S2). Heritability estimates for agronomic traits varied from 0.78 to 0.90 (Supplementary Table S3). Grain yield ranged from 13 kg ha^−1^ to 3299 kg ha^−1^ with an average of 1580 kg ha^−1^. The Jaccard’s dissimilarity matrix based on the agronomic data revealed the presence of large genetic variability among the inbred lines, with the highest genetic distance (0.81) observed between an IWDS inbred line and a TZLC derived inbred line whereas the lowest genetic distance (0.03) was observed between an IWDS line and a line derived from a composite.

**Figure 1:**
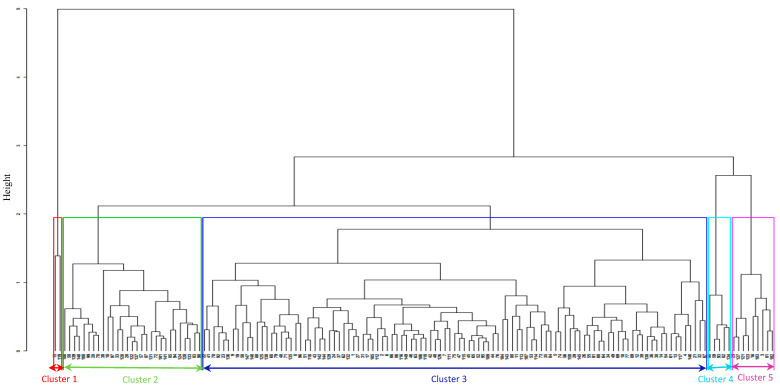
Hierarchical cluster analysis based on agronomic traits measured under Striga infestation condition using ward.D2 method showing the genetic relationships among the 150 inbred lines based on Gower’s dissimilarity matrix.

Assessment of the phenotypic diversity using the agronomic traits grouped the inbred lines into five clusters based on their reaction pattern to *S. hermonthica* (Figure 1). Cluster I consists of a known *Striga* susceptible line (5057) and a line derived from IWDS that supports the largest number of emerged *Striga* plants. The two inbred lines in this cluster also had the highest *Striga* damage symptom rating at 8 and 10 weeks after planting. Cluster II consists of twenty-nine inbred lines that support few emerged *Striga* plants, with moderate *Striga* damage symptoms and they produced intermediate grain yield. Inbred lines in this cluster are characterized as tolerant to *Striga* infestation. Cluster III consists of one hundred and five inbred lines that support fewer emerged *Striga* plants with varying levels of *Striga* damage symptoms. These lines are characterized as moderately resistant to *Striga* infestation. Cluster IV consists of five maize inbred lines with high *Striga* emergence count and damage symptoms. These lines yielded more than the susceptible lines and they are characterized as moderately susceptible to *Striga* infestation. Cluster V had nine inbred lines with low *Striga* emergence and *Striga* damage symptoms. These lines are characterized as resistant to *Striga* infestation.

The results of principal component analysis (PCA) showed that the first three components together accounted for 81% of the total phenotypic variation (Table 2). The first principal component explained 49% of the total phenotypic variation with the main contribution from seven variables. Principal component two accounted for 18% of the total variation with traits including days to silking and anthesis-silking interval contributing the most to this component.

**Figure 2:**
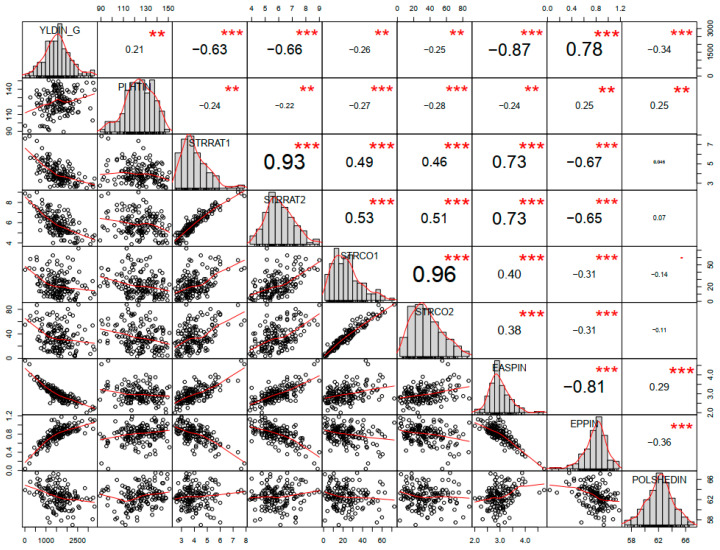
Correlation coefficient under *Striga* infested. *Striga* Damage Symptom Rating (STTRAT 1 and 2) = *Striga* damage rating at 8 and 10; Weeks After Planting (WAP), *Striga* Count or Emerged *Striga* plant (STRCO 1 and 2) = *Striga* count at 8 and 10 WAP, Plant Height Infested (PLHTIN), Ear Per Plant Infested (EPPIN), Ear Aspect Infested (EASPIN), Days to anthesis infested (POLSHEDIN), Grain Yield under Striga Infestation (YLDIN). *** *p* significant at 0.001, ** *p* significant at 0.01.

Further correlation analysis found a significant and positive correlation between grain yield and ear per plant and also between ear aspect and *Striga* damage rating at 8 and 10 weeks after planting. In contrast, there was a negative correlation between grain yield and ear aspect, *Striga* damage rating at 8 and 10 weeks after planting, and *Striga* count at 8 and 10 weeks after planting (Figure 2).

### 4.2. Genetic Diversity and Population Structure Based on SNP Markers

The SNP markers used in this study were unequally distributed across the ten maize chromosomes. The highest number of SNPs was found on chromosome 1, with 2532 SNPs, while the lowest was on chromosome 10, with 1208 SNPs. The summary statistics of the 150 inbred lines using the 16,735 SNP markers shows that the minor allele frequency varied from 0.05 to 0.50, with an average of 0.24. The observed heterozygosity rate ranged from 0.00 to 0.98 with an average of 0.10, however, most of the inbred lines (96%) had 1% heterozygosity rate, which is the expected amount of residual heterozygosity in inbred lines. The expected heterozygosity, also known as gene diversity, varied from 0.10 to 0.50, with an average of 0.32. Polymorphic information content values ranged from 0.10 to 0.47, with an average of 0.26 (Table 3).

**Figure 3:**
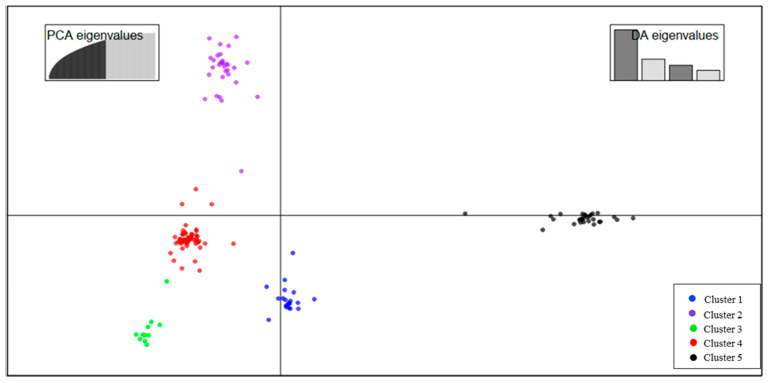
Discriminant analysis of principal component (DAPC) using 16,735 SNP markers. The axes represent the first two linear Discriminant. Each color represents a cluster, and each dot represents an inbred line. Each color represents the different subpopulations identified by DAPC analysis.

The population structure analysis performed through Admixture revealed a rapid elbow at *k* = 5, which grouped all the 150 inbred lines into five clusters (Supplementary Figure S1B). In addition, using the Bayesian information criterion (Supplementary Figure S1A), the optimal number of clusters was obtained at *k* = 5, which corresponds to the number of the cluster under the DAPC (Figure 3). Estimation of the cluster membership revealed that cluster IV had the highest number of inbred lines (54), and cluster III had the lowest number of inbred lines (8). The genetic distance between pairs of the 150 inbred lines ranged from 0.02 to 0.41, with an average of 0.33. The highest genetic distance (0.41) was observed between an early maturing inbred line (TZEC) and an intermediate maturing inbred line (IWDS) that combines resistance to *S. hermonthica* with tolerance to drought. The lowest genetic distance (0.02) was observed between two inbred lines showing low emerged *Striga* plants.

**Figure 4:**
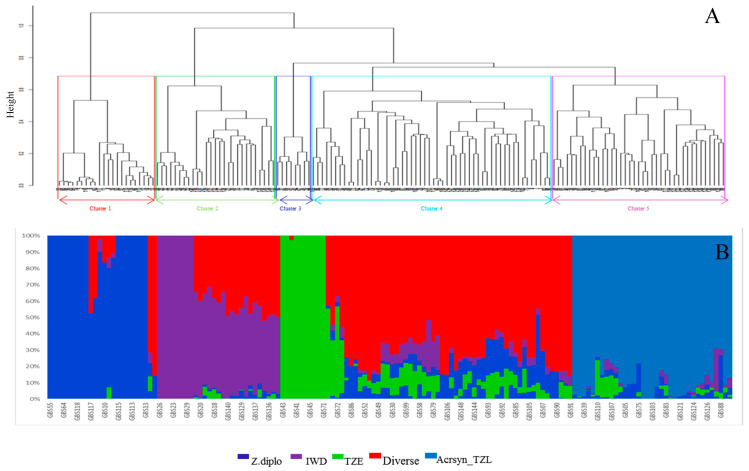
(**A**) Hierarchical cluster analysis based the genotypic data using ward.D2 method showing the genetic relationship among 150 maize inbred lines based on IBS using 16,735 SNPs. (**B**) Admixture plot showing clustering of the inbred lines into five clusters based on the cross-validation error and Bayesian-based clustering analysis. A vertical bar represents each inbred line. The colored sections in a bar indicate membership coefficients of the inbred line in the different clusters. Identified subgroups are cluster 1.

Based on the dissimilarity matrix generated from the SNPs, at a cophenetic coefficient correlation of 0.70 for the ward.D2, the inbred lines were grouped into five clusters (Figure 4A) based on their parental genetic background. Cluster I consists of 22 inbred lines, with 19 of them originating from a backcross containing ZDIP and the remaining three originating from TZLC. All inbred lines in this group had white kernel color, they are also high yielding and support few emerged *Striga* plants. Cluster II consists of 27 inbred lines derived from an intermediate maturing synthetic (IWDS). Most of the lines in this cluster are high yielding and support fewer emerged *Striga* plants. Cluster III consists of eight inbred lines, with seven of them originating from an early maturing composite (TZEC) and the remaining from a late-maturing synthetic (TZLC). Inbred lines in cluster II and III all had a white kernel color (Supplementary Table S4). Cluster IV had 54 inbred lines, with varying levels of resistance to *S. hermonthica* as well as a well-known susceptible line. Most of the inbred lines in this cluster originated from the different source populations. Cluster V consists of 39 inbred lines derived from the late maturing synthetic TZLC. Most of the lines originating from TZLC, they are high yielding and supported few emerged *Striga* plants (Figure 4A).

### 4.3. Combined Analysis of Phenotypic and Genotypic Data

**Figure 5:**
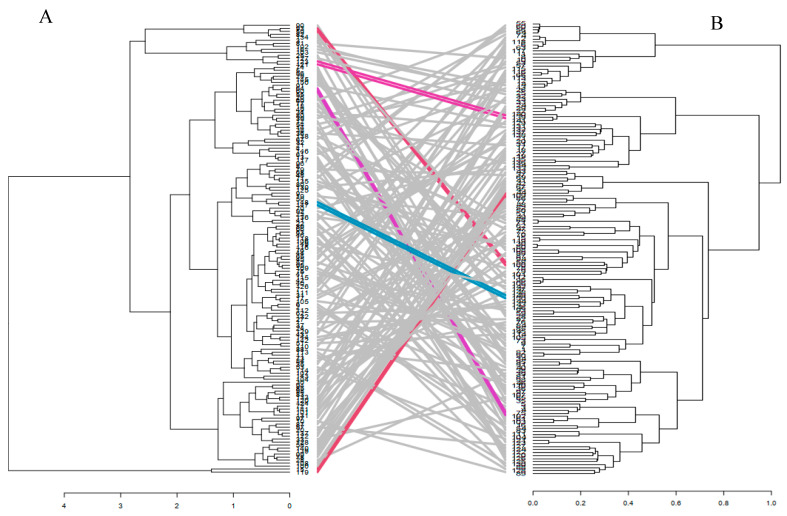
Comparison of hierarchical cluster analysis from the phenotypic under *Striga* infestation (**A**) and the genotypic (**B**). The ash lines in between the two dendrograms represent mismatched inbred lines from the genotypic to the phenotypic while the colored lines are inbred in the same position from phenotypic to the genotypic cluster.

**Figure 6:**
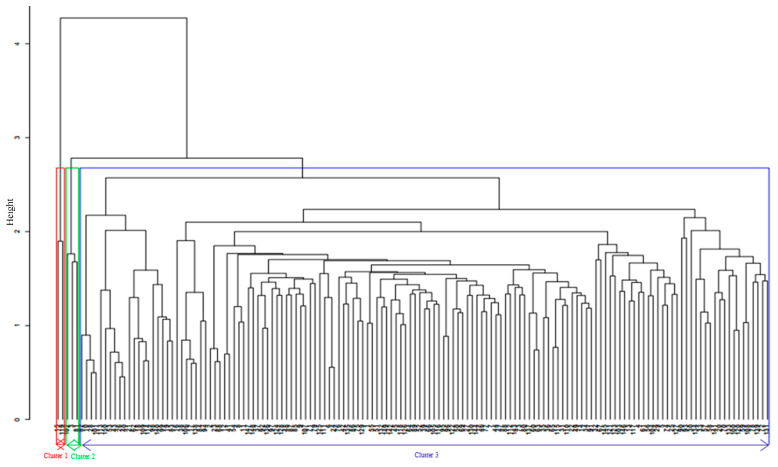
Hierarchical cluster analysis based on combined agronomic and molecular data using ward.D2 method.

The agronomic trait-based hierarchical cluster generated under *Striga*-infested condition was compared with the genotypic-based cluster analysis. It was observed that the clustering pattern of the inbred lines was different for both agronomic trait-based and the genotypic cluster. However, few inbred lines seven (5%) maintained the same group across both hierarchical clusters (Figure 5). These inbred lines are from diverse source populations with different reactions to *Striga* infestation. Six of the lines are from the ZDIP and the TZLC groups while the last line is a known susceptible inbred line. Membership and grouping patterns also changed between the agronomic-trait-based and molecular-based analyses. Genetic diversity assessment using the combined agronomic-trait-based and genotypic matrices (joint matrix) showed that the inbred lines clustered into three distinct groups (Figure 6). Cluster I consists of two susceptible and low-yielding inbred lines, whereas cluster II consists of three high yielding and resistant lines that support few emerged *Striga* plants. Cluster III consists of 145 inbred lines, with most of them having varying resistance reactions to *S. hermonthica,* but none of them was susceptible to *Striga*. Further analysis using the Mantel test found a very low correlation (*r* = 0.001) between the agronomic and the molecular dissimilarity matrices. However, a high correlation (*r* = 0.85) was observed between the joint matrix and the agronomic dissimilarity matrix, while a moderate correlation (*r* = 0.50) was observed between the joint matrix and the genotypic data-based matrix (Supplementary Figure S2).

## 5. Discussion

Analysis of variance under *Striga* infestation showed that there was significant variation among the inbred lines used in this study. The significant difference observed across environments and genotype X environment interaction for most of the traits measured could be due to seasonal factors [54,55]. The hierarchical cluster analysis based on agronomic characteristics under *Striga* infestation grouped the inbred lines according to their reaction pattern to *S. hermonthica,* with most of the inbred lines in each group emanating from different source populations. Cluster I comprises inbred lines that are susceptible to *Striga* infestation while cluster IV and II contains inbred lines that are tolerant to *Striga* infestation. Inbred lines in these two clusters yielded more than the susceptible lines but support the emergence of *Striga* plants and this can increase the *Striga* seed bank in the soil. Cluster III and V consist of inbred lines that are resistant to *Striga* infection; they support few emerged *Striga* plants, less *Striga* damage symptom rating, and were high yielding. Inbred lines in these two clusters can serve as parental lines in developing durable *Striga*-resistant maize hybrids and synthetics. Several studies have reported the importance of phenotypic traits in unraveling the diversity and differentiation in maize [25,56].

Phenotypic characterization is essential in describing breeding lines and also serves as meaningful criteria for selecting materials with desirable traits for breeding purposes. However, phenotypic traits are easily influenced by environmental factors. The advent of the molecular marker technique has made the evaluation of genetic diversity easier because environmental factors do not control them. Several types of these markers have been used to assess the level of genetic diversity in different crops ranging from dominant to codominant markers. For this study, 16,735 SNP markers from the genotyping-by-sequencing (GBS) platform were used to assess the genetic diversity and population structure of the inbred lines. The three complementary approaches used to define the optimal number of groups in this study (DAPC, hierarchical cluster analysis, and Admixture ancestry) all grouped the inbred lines into five distinct groups. The assigning of group membership in DAPC is in agreement with the hierarchical cluster analysis; this finding is consistent with reports from other studies [36,57,58]. The high cophenetic correlation coefficient (>0.70) observed for the hierarchical cluster analysis indicates the reliability of this approach to summarize the information of dissimilarity matrices [34]. Moderate levels of admixture were observed among the inbred lines used in this study, this indicate shared ancestry among the inbred lines. Most of the inbred lines used in this study emanated from the hybridization of various source populations that have been subjected to improvement under *Striga* infestation [15] and these source populations have different genetic backgrounds and exhibit broad genetic diversity.

The combined dissimilarity matrix of the agronomic-trait-based and molecular marker data grouped the inbred lines into three distinct clusters based on their reaction pattern to *S. hermonthica.* Seven inbred lines maintained the same cluster positions across both hierarchical clusters when compared with each other. The six inbred lines from the ZDIP and the TZLC groups are high yielding and support low emergence of *Striga* plants; they also have low *Striga* damage rating. These lines can be used as desirable parents to broaden and diversify the genetic base in *Striga* resistance breeding programs. The last inbred line is a known susceptible inbred line.

The low correlation observed between the genotypic and agronomic-trait-based distance matrices in this study should not be regarded as a limitation to access the genetic diversity but as an indicator of the complementarity of these methods [59]. The low correlation observed can also be attributed to the ability of the molecular marker to detect variations at the genetic level and they are not liable to natural or artificial selection unlike phenotypic markers [60]. In addition, molecular markers are selectively neutral, whereas the portion of the genome associated with the phenotypic trait is usually subjected to selection under the environmental influence [61,62]. Several authors have suggested that the best way to identify divergence among accessions is the combined use of molecular and phenotypic data, as these tools are complementary [33,34,35,36,37,60,61]. Andrade et al. [34] assessed the genetic diversity among sweet potato genotypes using morphological and molecular data. The study observed a low correlation between the distance matrix obtained with morphological and molecular data and this corroborates with the findings from our study.

The high and moderate correlation observed between the combined dissimilarity matrix and the phenotypic and genotypic dissimilarity matrices, respectively, is an indication that genetic diversity analysis based on the joint matrix is an invaluable tool to cumulatively and reliably allocate genotypic and phenotypic information. This result is consistent with the findings of other studies [36,61,62]. The use of both agronomic trait and molecular data in assessing genetic diversity helps to maximize genetic diversity as well as productivity in crop plants [63]. Studies have also shown the efficiency of combined dissimilarity matrix of morphological and molecular data in deciphering the genetic variability in different crops [33,34,64].

In conclusion, assessment of the genetic diversity using both agronomic traits and molecular data revealed the existence of considerable genetic variability among the evaluated materials. It also provides invaluable information on the reaction patterns of the inbred lines to *S. hermonthica*. The molecular diversity analysis was able to group the inbred lines based on their genealogy and historical background than the phenotypic diversity analysis. However, the grouping patterns between both clusters were inconsistent due to non-overlapping information between the data types. The joint diversity analysis explores the synergy of the two approaches (molecular and agronomic) by capturing the information to provide a comprehensive understanding of the entire diversity among the inbred lines.

## Figures and Tables

**Table 1:** List of source populations for the 150 inbred lines used in this study.

Source Population	Genetic Background of Source Population	Lines Evaluated
ZDIP	Lines derived from a backcross (BC4) containing a *Z. diploperennis* accession as a donor parent plus lines derived from bi-parental crosses involving one parent derived from the same *Z. diploperennis* backcross	39
TZLC	A late-maturing composite formed by crossing TZB-SR with seven inbred lines developed for field resistance to *S. hermonthica* plus lines derived from bi-parental crosses involving one parent derived from the same source populations	44
TZEC	An early maturing composite formed by crossing TZESR-W C3 with eight inbred lines developed for field resistance to *S. hermonthica* plus lines derived from bi-parental crosses involving one parent derived from the same composite	13
IWDS	A synthetic formed from medium maturing white inbred lines and improved for resistance to *Striga* and drought plus lines derived from bi-parental crosses involving one parent derived from these synthetic	30
MIXED	Lines derived from diverse source populations plus the tolerant lines expensively used as donors of field resistance to form populations	24

**Table 2:** Principal component analysis and variables contributing the most under *Striga* infestation.

Variables	PC1	PC2	PC3
Days to silking	−0.441	0.855	−0.068
Ear aspect	0.884	0.070	−0.303
Ear per plant	−0.824	−0.064	0.324
Plant height	−0.388	0.212	−0.221
*Striga* count at 8 WAP	0.651	0.148	0.712
*Striga* count at 10 WAP	0.633	0.182	0.719
*Striga* damage at 8 WAP	0.878	0.107	−0.092
*Striga* damage at 10 WAP	0.881	0.201	−0.085
Yield	−0.786	−0.173	0.434
Anthesis silking interval	−0.302	0.908	0.014
Eigenvalue	4.886	1.745	1.478
Variance (%)	48.863	17.459	14.784
Cumulative variance (%)	48.863	66.321	81.105

Values in bold indicate the most relevant character (˃0.400) that contribute to the variation of the component.

**Table 3:** Diversity indices of the 150 inbred lines based on 16,735 single-nucleotide polymorphism (SNP) markers.

	MAF	Ho	He	PIC
Minimum	0.05	0.00	0.10	0.1
Maximum	0.50	0.98	0.50	0.47
Mean	0.24	0.10	0.32	0.26

MAF = minor allele frequency, H_O_ = observed heterozygosity, H_e_ = expected heterozygosity, PIC = polymorphic information content.

## Supplementary Materials

The following are available online at https://www.mdpi.com/2223-7747/9/9/1223/s1, Figure S1: Graph (**A**) Bayesian information criterion versus the number of clusters and (**B**) cross-validation error versus K value, Figure S2: Mantel correlation test performed between the different dissimilarity matrices alongside the combined matrix. Sim = similarity coefficient, beacon = Mantel threshold indicating the correlation, Table S1: Traits measured, their description and unit, Table S1: Mean squares from the combined ANOVA for traits recorded under infestation condition for 2 years evaluated in 2 locations, Table S3: Mean and standard errors of variables under Striga infestation and Striga non-infestation across the entire four environments, Table S4: Grouping of the 150 inbred lines using 16,735 SNPs.

## Abbreviations

ANOVA	Analysis of variance
BIC	Bayesian information criterion
CTAB	Cetyltrimethylammonium bromide
DAPC	Discriminant analysis of principal components
DNA	Deoxyribonucleic acid
GBS	Genotyping-by-sequencing
IBS	Identity by State
LD	Linkage disequilibrium
SNP	Single nucleotide polymorphism
SSA	sub-Saharan Africa
PIC	Polymorphic information content
TASSEL	Trait Analysis by aSSociation, Evolution and Linkage
