# Supplementary material for: Genetic Diversity and Population Structure of Maize Inbred Lines with Varying Levels of Resistance to Striga hermonthica Using Agronomic Trait-Based and SNP Markers
Source: Plants (Basel). 2020 Sep 17;9(9):1223. doi: 10.3390/plants9091223 (PMC7570130; doi:10.3390/plants9091223)

Supplementary Table S1. Traits measured, their description and unit

S/N	Traits	Traits Abb.	Trait description	Unit
1	Days to anthesis	Anthesis	The number of days from planting to the time when 50% of the plants have anther shedding pollen	Count
2	Days to silking	D.S.	The number of days from planting to the time when 50% of the plants have emerged silk	Count
3	Anthesis silking interval	ASI	The interval in days between dates of silking and anthesis	Count
4	Plant height	PLHT	The distance from the base of the plant to the height of the first tassel branch	cm
5	Ear aspect	EASP	It is scored on a scale of 1-5 , where 1= clean, uniform , large and well-filled ears and 5 = rotten small partially filled ear	Scale
6	Plant aspect	PASPUN	It is scored on a scale of 1-9 , where 1= clean, uniform , large and well-filled ears and 9 = rotten small partially filled ear	Scale
7	Ear per plant	EPP	Total number of ears with at least one fully developed grain divided by the number of harvested plants	Count
8	*Striga* emergence count	STRCO 1 & 2	Number of emerged striga plant per plot at 8 and 10 weeks after planting [WAP]	Count
9	*Striga* damage rate	STRRAT 1 & 2	It is scored on a scale of 1-9 , where 1= no visible host plant damage symptom and 9 = all leaves are completely scorched and finally dead plants. Taken at 8 and 10 WAP	Scale
10	Grain yield	Yield/G.Y.	Calculated from ear weight and grain moisture	kg

Supplementary Table S2. Mean squares from the combined ANOVA for traits recorded under infestation condition for 2years evaluated in 2 locations

Source	Df	Plant height	Ear Aspect	Ear per plant	Striga rate 8 WAP	Striga rate 10 WAP	Emerged Striga 8 WAP	Emerged Striga 10 WAP	Grain Yield [kg ha^{-1}]
**Geno**	149	1347.57§	1.27§	0.25§	7.04§	7.65§	1529.72§	2433.63§	3029370.5§
**Env**	3	24923.75§	7.35§	4.26§	174.40§	427.67§	5672.25§	39063.45§	55554413.3§
**Rep[Env]**	4	1542.45§	1.18§	0.19§	1.08	2.86**	91.15	282.23	2934078.5§
**Blk[Rep*Env]**	112	305.96§	0.32§	0.06§	1.07§	1.40§	454.29§	1117.56§	685188.6§
**Geno*Env**	446	140.25***	0.29§	0.03	1.18§	1.24§	269.12***	531.70**	546418.6§
**Error**	483	106.25	0.18	0.03	0.63	0.72	202.1	421.76	356115
**CV[%]**		8.26	14.22	22.25	19.62	14.35	60.95	58.84	33.28

*Significant at $p \leq 0.05$ level,
**Significant at $p \leq 0.01$ level,
***Significant at $p \leq 0.001$ level,
§Significant at $p \leq 0.0001$ level respectively. Blk = Block, Env= Environment, Rep = Replication, CV = Coefficient of Variation, WAP = weeks after planting. ns = data not significant

Supplementary Table S3. Mean and standard errors of variables under *Striga* infestation and Striga non-infestation across the entire four environments

**Variable**	**Min**	**Max**	**Mean**	**Std error**	**Heritability**
Grain yield infested	13.00	3299	**1580**	270.5	0.83
Plant height (cm)	89.25	151	124.75	4.25	0.90
Ear per plant (number)	0.00	1.20	0.78	0.06	0.88
Ear aspect (1-5)	2.00	4.75	3.00	0.19	0.78
Striga damage rating 8 WAP	3.00	8.00	4.05	0.39	0.85
Striga damage rating 10 WAP	4.00	9.00	5.91	0.4	0.85
Emerged Striga count 8 WAP	1.00	73.00	23.31	5.85	0.85
Emerged Striga count 10 WAP	3.00	88.00	34.88	8.29	0.8

WAP=weeks after planting

Supplementary Figure S1: Graph [A] Bayesian information criterion versus the number of clusters and [B] cross-validation error versus K value

A

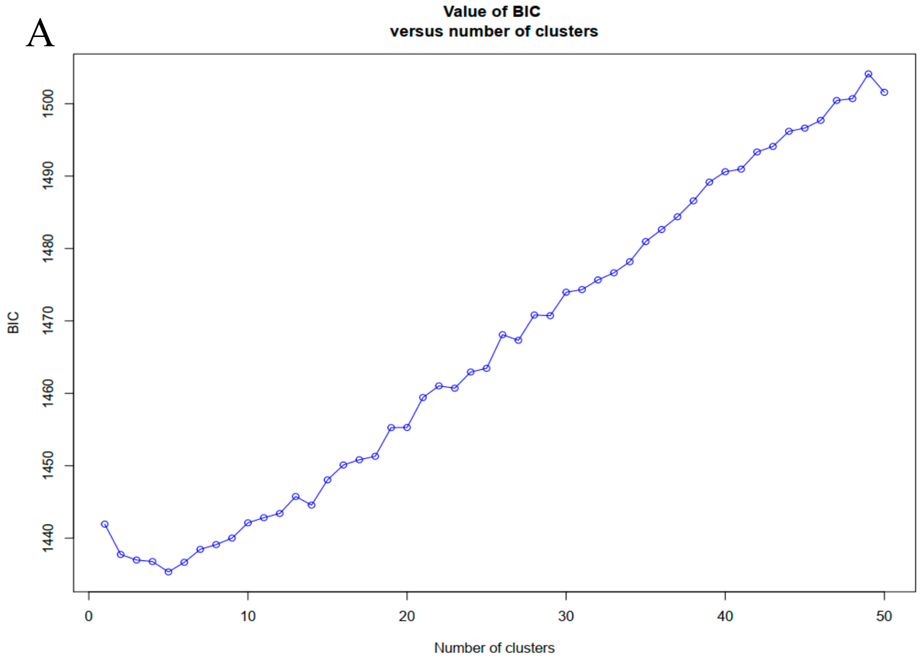

B

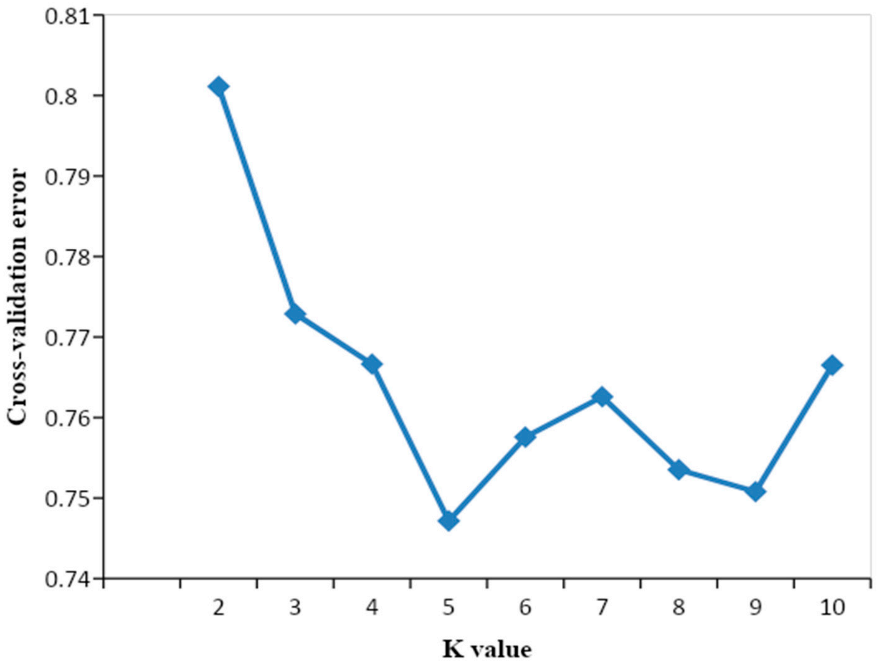
0.81
0.8
0.79
0.78
0.77
0.76
0.75
0.74
Cross-validation error
2
3
4
5
6
7
8
9
10
K value

Supplementary Table S4. Grouping of the150 inbred lines using 16,735SNPs

Groups	Inbred lines	Grain color	Source population
1	22	White	ZDIP
2	27	White	IWDS
3	8	White	TZEC
4	54	White and Yellow	Mixed
5	39	White and Yellow	TZLC

Supplementary Fig S2: Mantel correlation test performed between the different dissimilarity matrices alongside the combined matrix. Sim = similarity coefficient, beacon = Mantel threshold indicating the correlation.

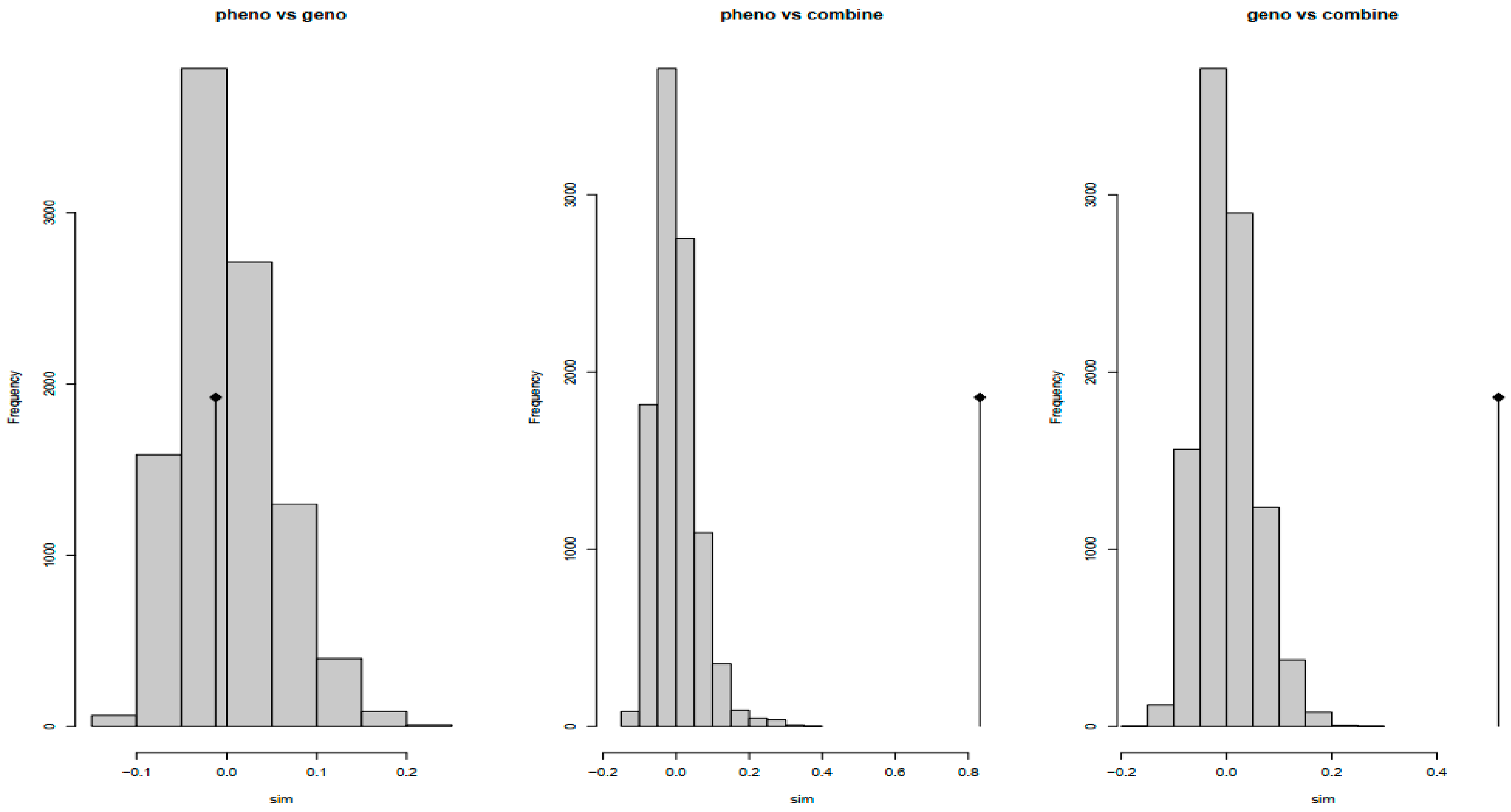

Supplement: Supplementary file 1 [file plants-09-01223-s001.pdf]
